# Synthesis of new magnetic nanocatalyst Fe_3_O_4_@CPTMO-phenylalanine-Ni and its catalytic effect in the preparation of substituted pyrazoles

**DOI:** 10.1038/s41598-023-29598-6

**Published:** 2023-02-13

**Authors:** Samaneh Bikas, Ahmad Poursattar Marjani, Sepideh Bibak, Hamideh Sarreshtehdar Aslaheh

**Affiliations:** Department of Organic Chemistry, Faculty of Chemistry, Urmia University, Urmia, Iran

**Keywords:** Catalysis, Green chemistry

## Abstract

In this study, a new, efficient and stable magnetically heterogeneous nanocatalyst of Fe_3_O_4_@CPTMO-phenylalanine-Ni via multi steps process starting from simple and cost-effective precursors was designed and successfully synthesized, and physico-chemical, structural, and magnetic properties have fully been characterized by several analytical methods involving SEM–EDS, FT-IR, TGA, VSM, XRD, ICP, BET, TEM, and EMA. The catalytic performance of the Fe_3_O_4_@CPTMO-phenylalanine-Ni can be used as an effective and recyclable nanocatalyst with facile separation by magnetic forces for the preparation of substituted pyrazoles with high yields through the one-pot, three-component condensation reaction of various arylglyoxals, diketones, and 1*H*-pyrazole-5-amines under mild conditions. The nanocatalyst’s activity after being used by four consecutive times in a cycle reaction without distinct deterioration remained unchanged or was found to be a slight decrease. The advantages of this study are simplicity, low cost, facile synthesis process, and environmentally secure nature.

## Introduction

Nanoparticles are known materials that have attracted significant attention due to their high surface area and easy retrieval. Heterogeneous nanocatalysts are easily separated from the reaction and have a high surface area due to the preparation of nanoparticles with different shapes and sizes; they have received particular attention^1,2^. Magnetic nanoparticles have a special place due to their advanced application in medicine, like some effective cancer treatments for cancer^3,4^. Nanoparticles containing therapeutic substances can target a specific organ so that it has few side effects^5–7^. Also, nanoparticles were extensively used to synthesize chemistry materials, catalysts, color pigments, etc. Especially Fe_3_O_4_ nanoparticles, considered for their easy separation at the end of the reaction, have received much attention^8–10^.

Polyethylene glycol (PEG) as an environmentally friendly flexible polymer and non–toxicity was utilized in protective coating for nanoparticles^5^. PEG is easily absorbed by magnetic nanoparticles and increases blood circulation^11,12^. Magnetic nanoparticles coated with PEG can resist protein absorption, and prevent the detection of macrophage cells^11,13^. Furthermore, they can detect malignant cancer cells^14^. Mentioned nanocatalysts are easily separated at the end of the reaction using an external field from reaction media^15,16^.

**Figure 1:**
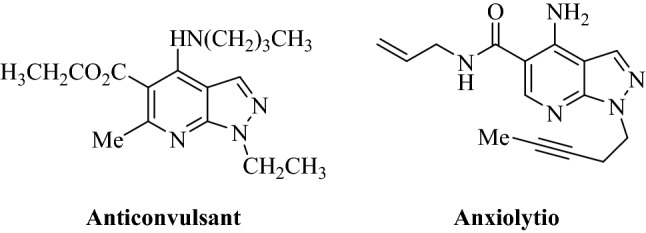
A few examples of fused pyrazoles with biological properties.

Today heterocycles as extraordinary compounds are widely presented and distributed in the structure of many natural substances with many applications such as hormones, vitamins, drugs, etc. The distinctive features of heterocycle substrates make their formation, a perpetual area of investigation^17^. In the context of introducing novel routes in organic synthesis, implementation and design of multi-component reactions (MCRs) in the production of various heterocyclic scaffolds have been accomplished prominence progress^18–21^. In this regard, pyrazole-containing heterocyclic scaffolds are a substantial category of nitrogen heterocyclic that exhibit various attractive pharmaceutical and remarkable biological activities, including anticancer, anti-bacterial, antiproliferative, anti-hyperglycemic, and anti-depressant^22–24^. Some examples of biologically effective pyrazoles are outlined in Fig. 1.

**Figure 2:**
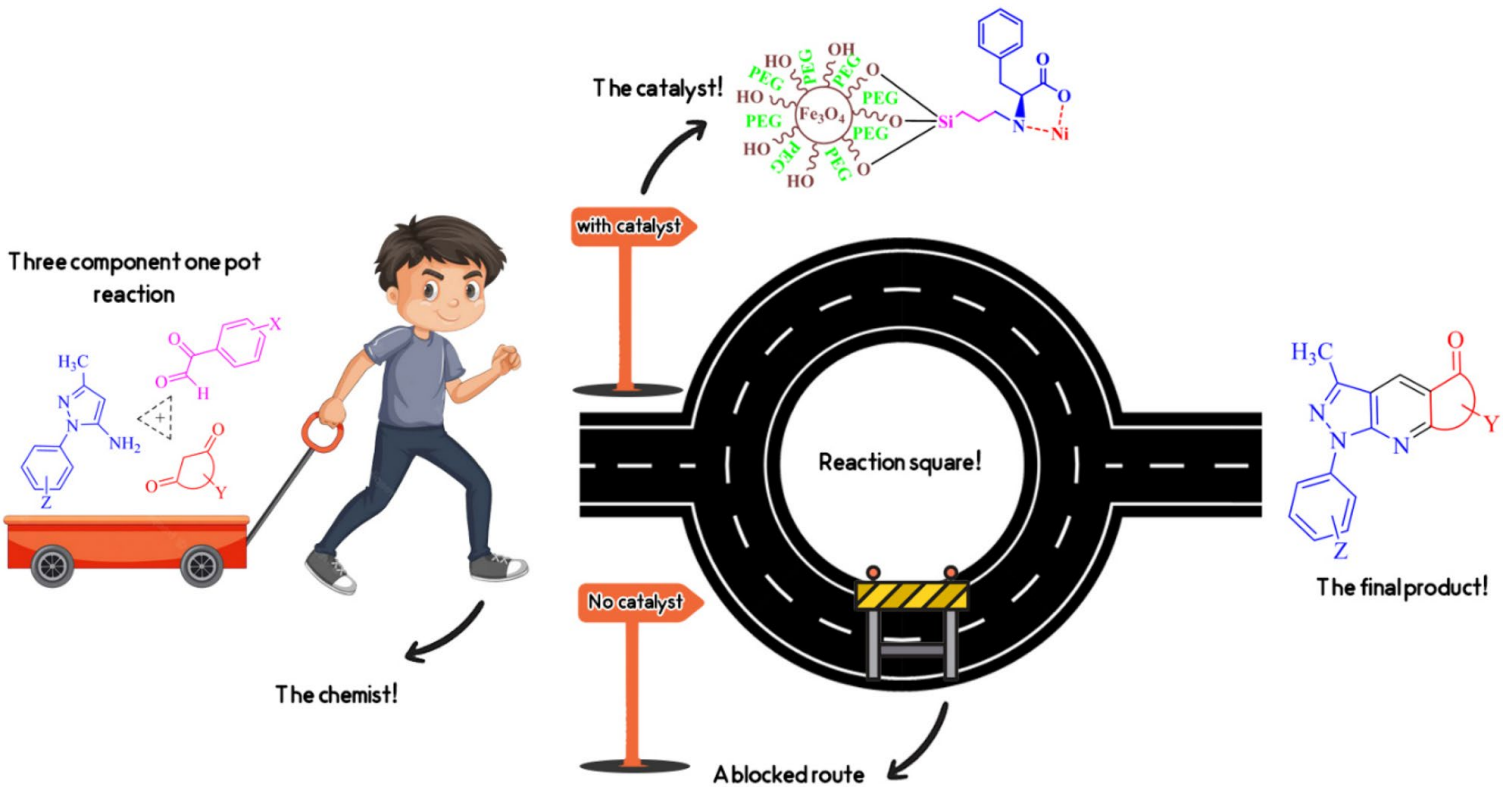
Synthesis of pyrazole derivatives with Fe_3_O_4_@CPTMO-phenylalanine-Ni as a catalyst. (Drawn by Photoshop 2015 and ChemDraw 20.1.1 Cliparts used from freepik.com).

In some literature, several routes have been elaborated for synthesizing pyrazole analogs^25,26^. However, these methods are restricted due to using multistep procedures, low yields^27^, long reaction time, rigid work-up, and also not economical^28^, Therefore, efforts to introduce a new procedure to furnish these compounds are vital. As part of continuing attempts in the field of catalysts^29–31^, in this work, the Fe_3_O_4_@CPTMO-phenylalanine-Ni as a new nanocatalyst was synthesized, and its application to the synthesis of pyrazole rings via one-pot, three-component reactions using arylglyoxals, cyclic ketones, and 1*H*-pyrazole-5-amines, under appropriate laboratory conditions, have been assessed (Fig. 2).

## Result and discussion

**Figure 3:**
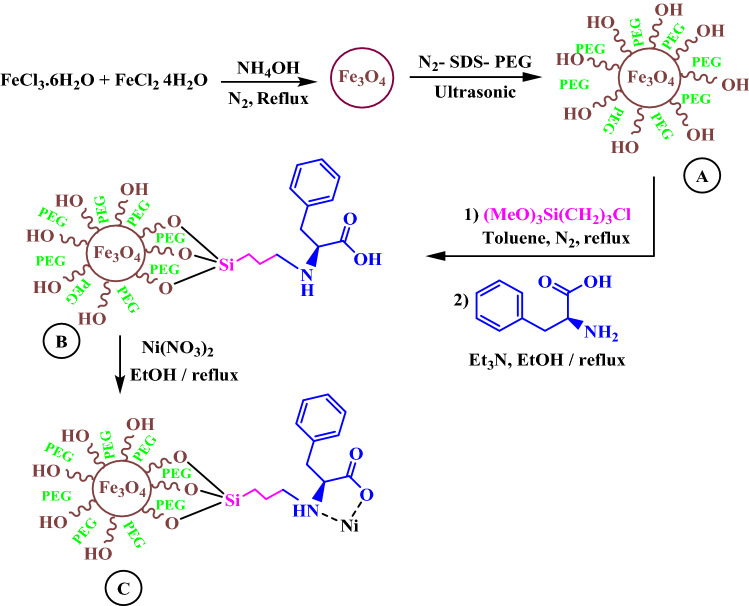
The stepwise synthesis pathway of Fe_3_O_4_@CPTMO-phenylalanine-Ni.

This research demonstrates the use of magnetic, recyclable, and reusable nanocatalysts in preparation of fused pyrazoles from cheap starting materials. The process synthesis of Fe_3_O_4_@CPTMO-phenylalanine-Ni via multi steps process represents in Fig. 3.

### Characterization

The nickel complex-supported MNP magnetic nanoparticles were determined by FT-IR, SEM–EDS, TGA, XRD, ICP, VSM, BET, EMA, and TEM analysis.

#### FT-IR analysis

**Figure 4:**
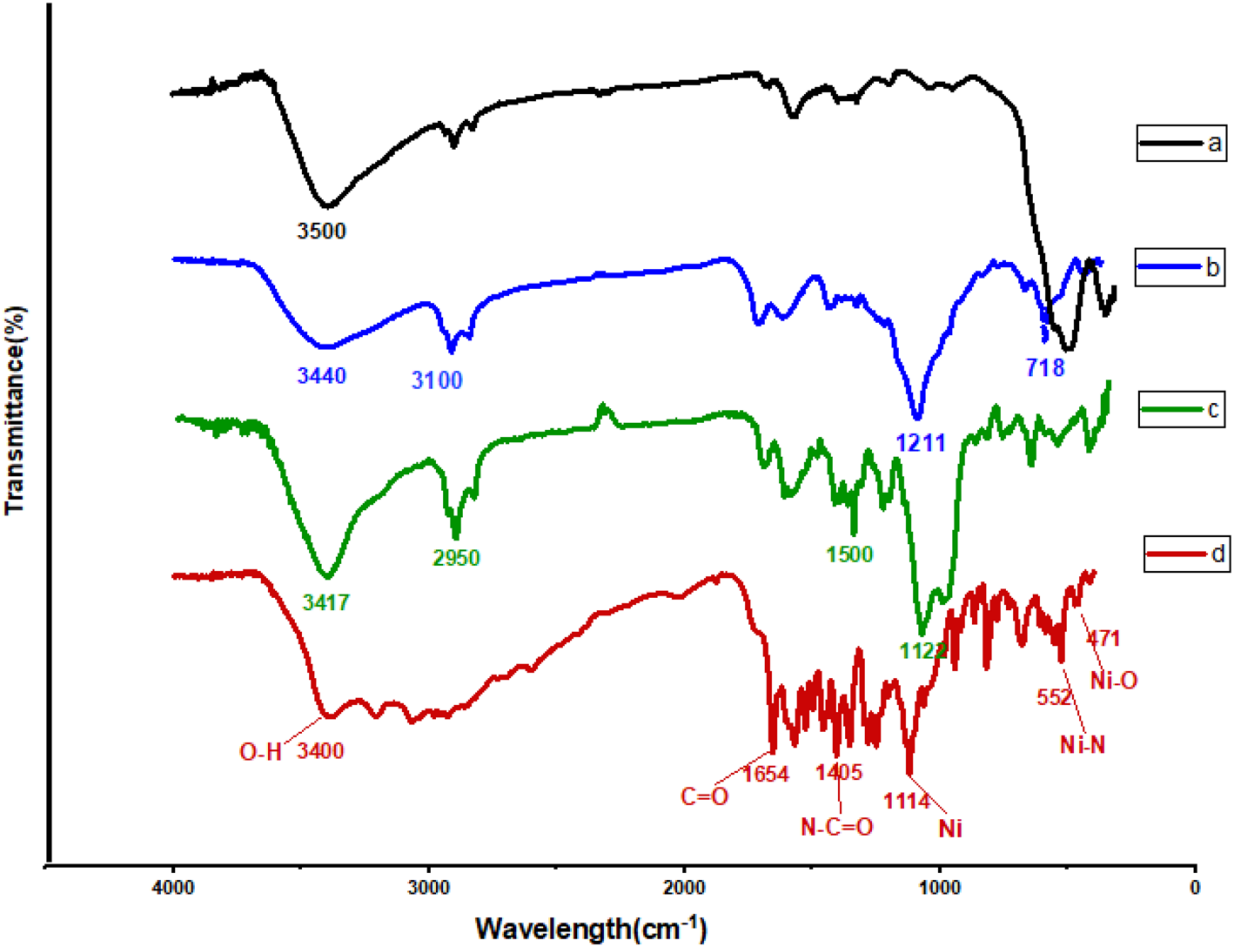
FT-IR spectra of Fe_3_O_4_ (**a**), Fe_3_O_4_@CPTMO (**b**), Fe_3_O_4_@CPTMO-phenylalanine (**c**), and Fe_3_O_4_@CPTMO-phenylalanine-Ni (**d**).

Using Fourier-transform infrared spectroscopy, the functional groups in the synthesized nanocatalyst can be discussed and investigated^32^. The FT-IR spectrum of the prepared nanoparticle is presented in Fig. 4. In the FT-IR spectrum of Fe_3_O_4_, the absorption peaks are observed near the 526, 1618, and 3500 regions, which correspond to the vibrational tensile frequency of group Fe–O, and O–H bending, and O–H stretching respectively (Fig. 4a). Due to the connection of PEG, absorption bands at 2955, and 718–1211 cm^−1^ represent the characteristic which is corresponding to aliphatic C–H stretching, Si–O stretching, and Si–O–Si symmetric and asymmetric stretching respectively (Fig. 4b). A peak at 3100 cm^−1^ is relevant to the C–H stretching modes of n-propyl that can correspond to symmetric/asymmetric tensile vibrations of the mesoporous structure of Si–O. The peaks in the region of 3417, 1122, and 1272 cm^−1^ correspond to vibrations of O–H stretching vibrations. In Fig. 4c, absorption peaks are observed near 1800 and 2950 cm^−1^ attributed to asymmetric/symmetric C=C tensile vibrations and confirming the successful linking of phenylalanine. Figure 4d, shows the FT-IR spectrum of Fe_3_O_4_@CPTMO-phenylalanine-Ni. The appearance of a broad peak at 1114 cm^−1^ corresponds to the connection of Ni metal. Also, the peaks are observed in 3400, 2981, 1654, 1405, 552, and 471 cm^−1^ regions which are related to O–H, C–H, C=O, N–C=O, Ni–N, and Ni–O connections, respectively.

#### SEM analysis

**Figure 5:**
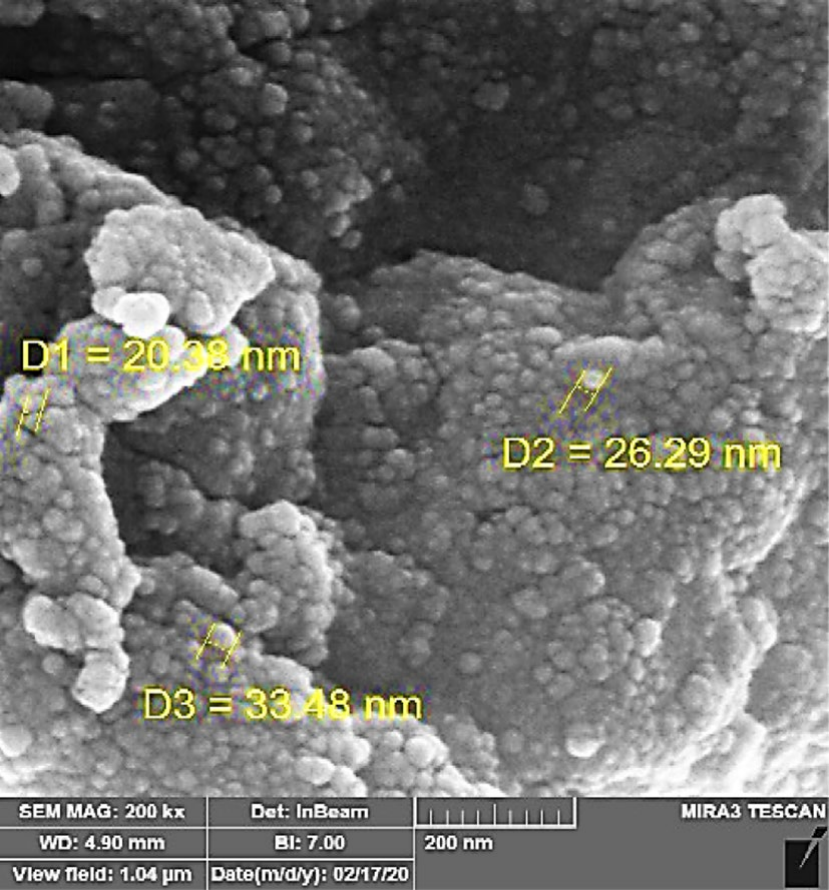
The SEM contains the size of Fe_3_O_4_@CPTMO-phenylalanine-Ni.

SEM analysis was used to determine the catalyst's particle size, morphology, and internal micro-structure particle^33^. Figure 5, illustrates the SEM images and the elemental analysis mapping of the synthesis nanocatalyst. In these images, morphological changes and particle size of the catalyst have been investigated. As seen in this Figure, the nanoparticle has regular and spherical morphology. These images show that the nanoparticle sizes ranged from 20 to 33 nm.

#### EDS and EMA (Elemental mapping analysis)

**Figure 6:**
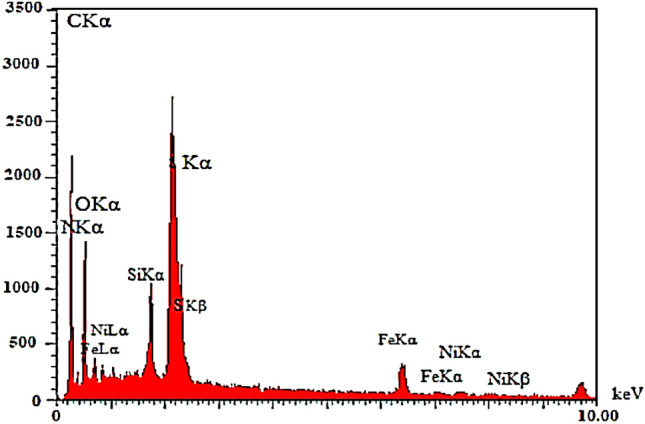
EDS analysis of Fe_3_O_4_@CPTMO-phenylalanine-Ni.

**Figure 7:**
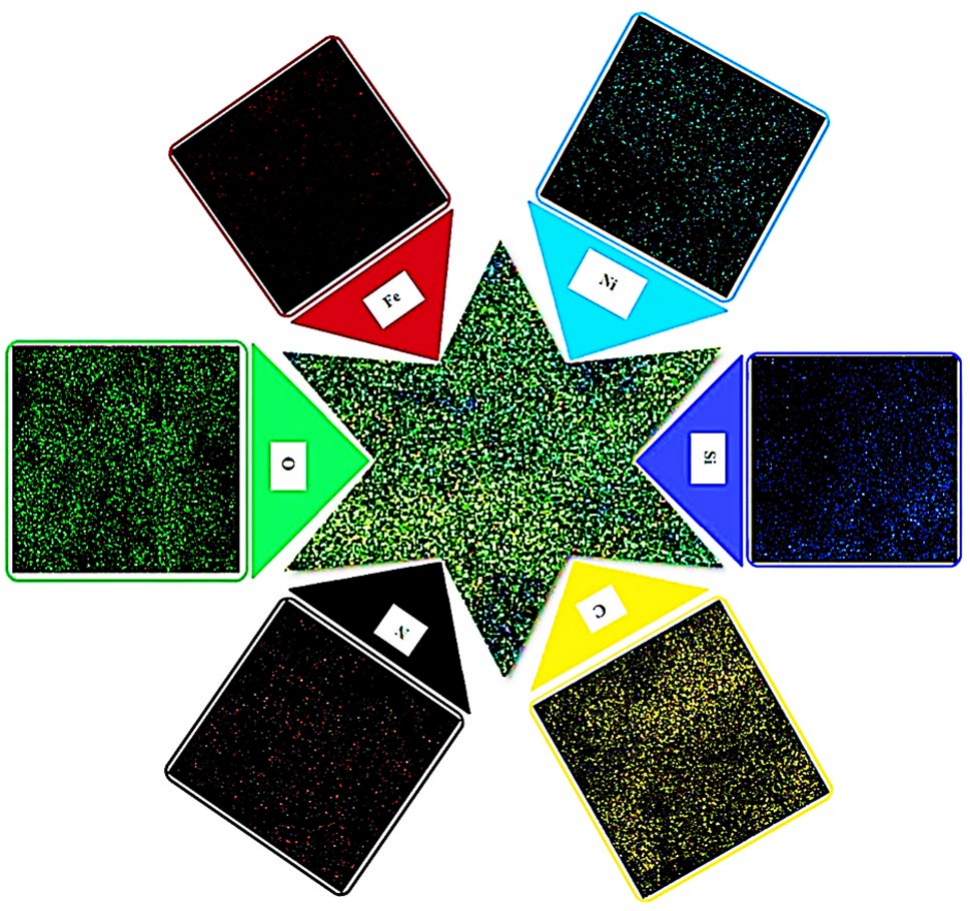
Elemental mapping analysis of Fe_3_O_4_@CPTMO-phenylalanine-Ni.

The EDS analysis was performed to show that supported nickel metal was on the MNPs surface (Fig. 6). The results show the attendance of carbon, nitrogen, oxygen, silicon, iron, and sulfur elements in functionalized MNP with a mass percentage of 40.63, 8.92, 38.34, 9.38, 0.50, and 2.22, respectively. The amount of Ni is 2.6% was specified through ICP-OES (Inductively Coupled Plasma-Optical Emission Spectrometry). The percentages of each element in the EDS analysis of Fe_3_O_4_@CPTMO-phenylalanine-Ni are presented in Table 1. Also, the acquired outcome from elemental mapping analysis affirmed the being of C, N, O, Fe, Ni, and Si, elements in the main structure of the nanocatalyst (Fig. 7).

**Table 1:** The percentages of each element in EDS analysis.

Element	C	N	O	Si	Fe	Ni
%W	49.76	9.37	35.26	4.91	0.13	0.56

#### TGA/DTA analysis

TGA analysis was accomplished to inquire about the effect of temperature on the nanocatalyst. The obtained curve is presented in Figure S1 (Supporting Information). This figure demonstrates the TGA diagram, which affirms the stability and attendance of the number of fixed categories on nanostructures. The first start of degradation and weight loss in all three curves **a-c** was fewer than 200 °C which corresponds to the rejection of absorbed solvent molecules and OH groups on the surface of the nanocatalyst. The second weight loss in curves **a** and** b** detected in the range of 200–400 °C is related to the removal of the phenylalanine ligand on the surface of the nanocatalyst. The three and greater loss weight in curves **a** and **c** detected in the region of 400–680 °C refers to degradation of the CPTMO group. The final weight loss in curve **a**, which occurs beyond 700 °C is attributed to the thermal decomposition of the nanostructure and confirms the stability of the synthesized catalyst at high temperatures. In addition, a weight loss of 31% was observed at about 200–700 °C.

#### VSM analysis

**Figure 8:**
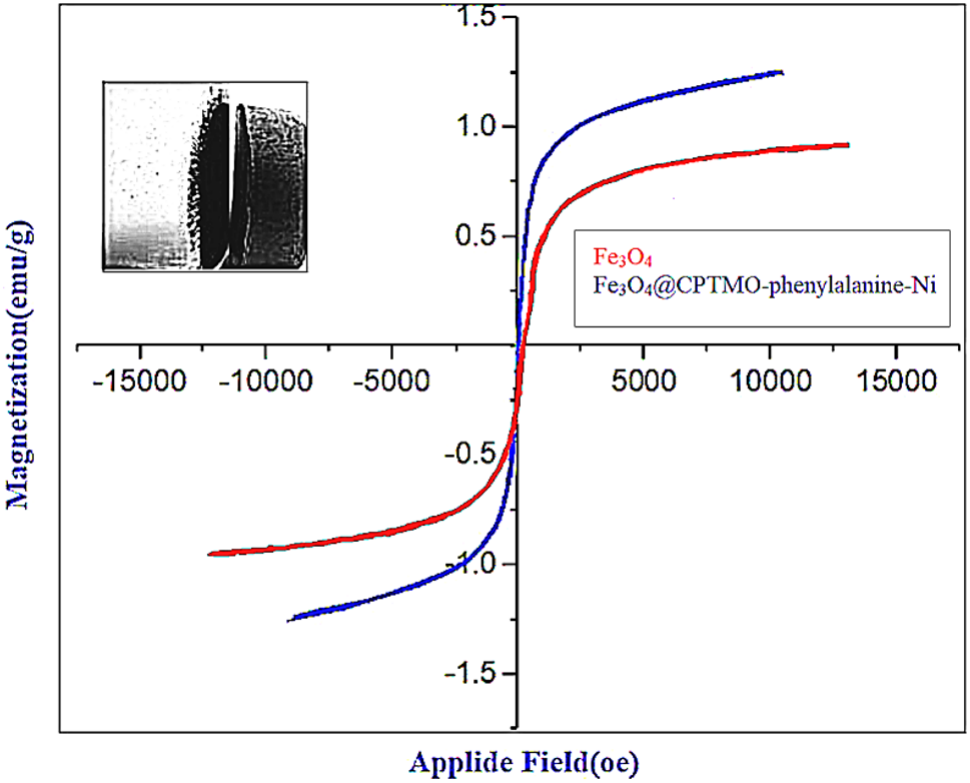
VSM data of Fe_3_O_4_@CPTMO-phenylalanine-Ni.

Vibrating sample magnetometry (VSM) examines the magnetic properties of particles. The VSM analysis was used to study the magnetic activities of the nanocatalyst, and the result is depicted in Fig. 8. According to chart data, the intensity of the saturation magnetization (MS) of Fe_3_O_4_@CPTMO-phenylalanine-Ni was 5 emug^−1^. Furthermore, magnetic particles have a high para magnetism due to no residual and zero forcible energy.

#### XRD analysis

**Figure 9:**
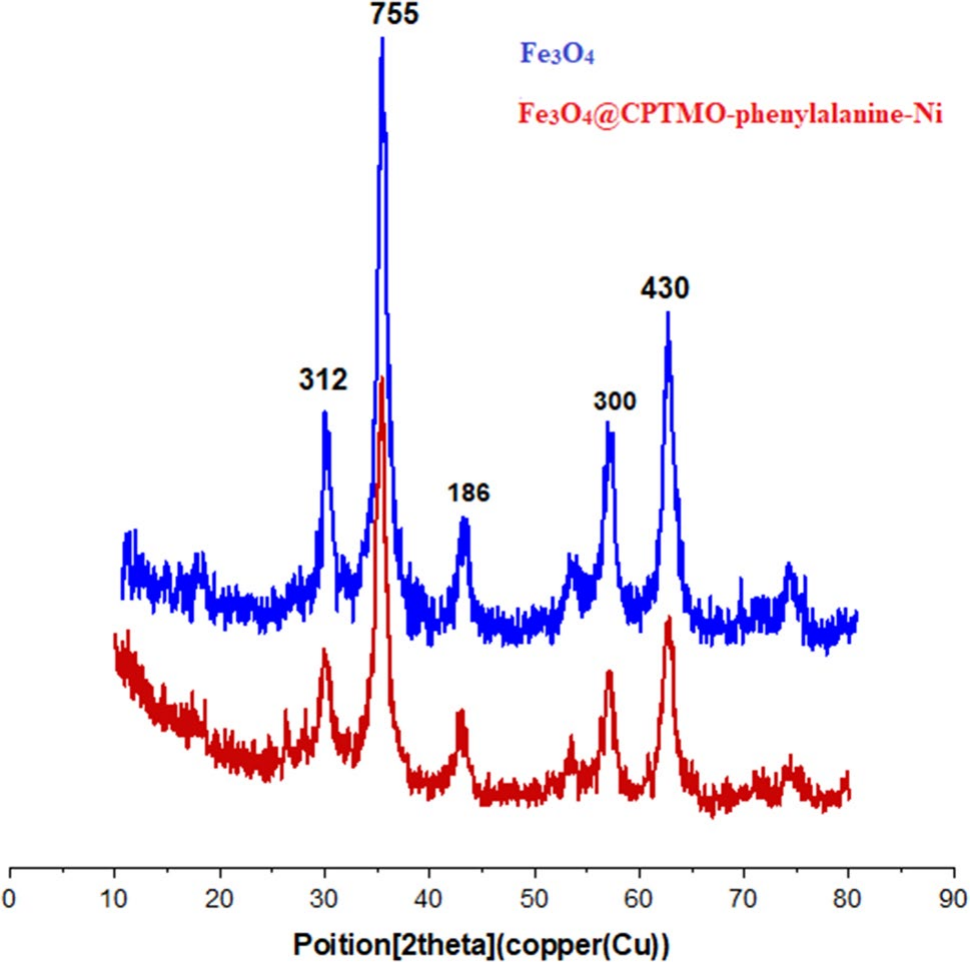
XRD pattern of Fe_3_O_4_ and Fe_3_O_4_@CPTMO-phenylalanine-Ni.

The crystalline nature of the nanoparticles was confirmed by XRD studies. The wide-angle XRD pattern was used to determine the number quantity of crystalline phases of prepared samples (Fig. 9). As shown in Fig. 9. strong reflections for Fe_3_O_4_ appeared at 2θ = 29.24°, 34.74°,49.19°, 56.24°, and 65.54° that relevant to the (312), (755), (186), (300), and (430) crystal planes of a pure Fe_3_O_4_ with a cubic spinel structure. All the peaks of Fe_3_O_4_ have appeared in the XRD pattern of Fe_3_O_4_@CPTMO-phenylalanine-Ni, also, appeared peaks were observed at 2θ = 29.2°, 29.45°, 30.00°, 36.65°, 42.65°, 57.45°, and 62.95°, related to orthorhombic crystal system of Fe_3_O_4_@CPTMO-phenylalanine-Ni (D = 44.5 nm).

The crystallite size data were calculated by the Scherrer equation:

$$D=\frac{K\uplambda }{\beta \frac{1}{2}\mathrm{cos}\theta }$$

#### Nitrogen-physical adsorption studies

The nitrogen adsorption/desorption isotherm and BET plots of the nanocatalyst are given in Figure S2 (Supporting Information). The specific surface area was assessed using the BET equation. Also, the pore size distribution curves were obtained using BJH analysis. The information obtained from the synthesized nanocatalyst shows that the pore size, pore volume, and surface area of the nanoparticle are 5.2734 nm, 0.7789 cm^3^/g, and 3.39 m^2^g^−1^, respectively. These results indicate that the Fe_3_O_4_@CPTMO-phenylalanine-Ni was acquired as a mesoporous type.

#### TEM analysis

**Figure 10:**
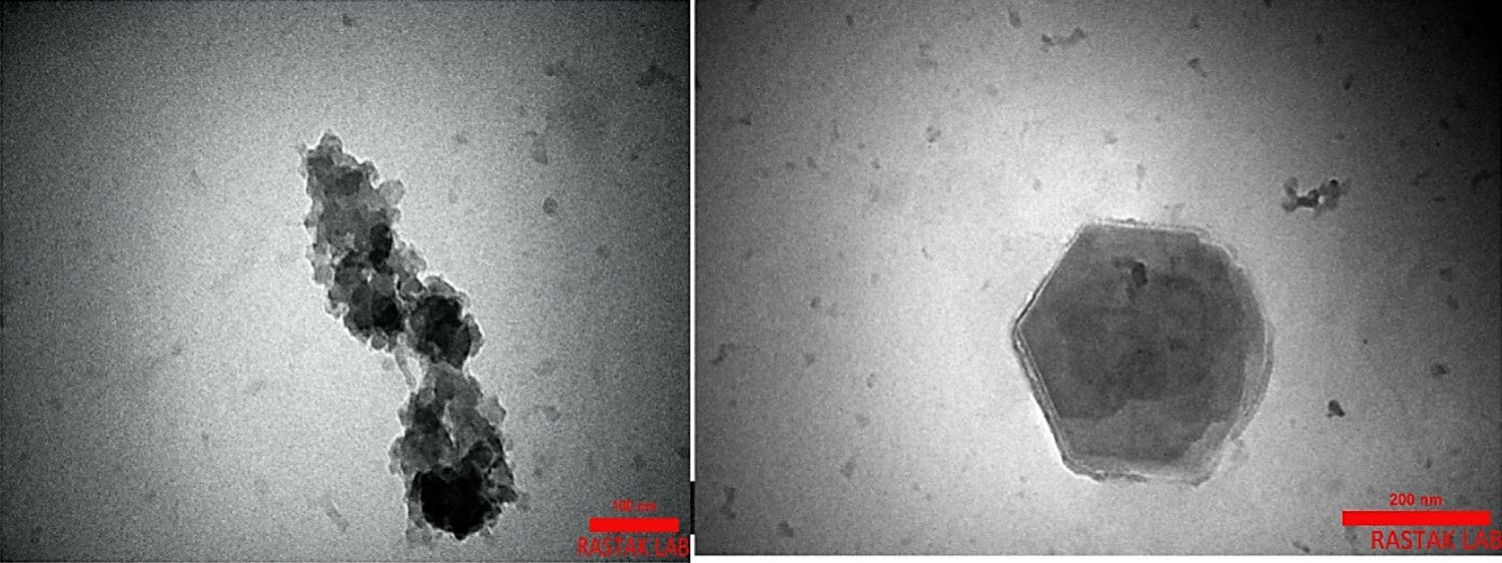
TEM analysis of Fe_3_O_4_@CPTMO-phenylalanine-Ni.

Figure 10, shows that TEM analysis; was used for more characterization of the surface morphology of the nanocatalyst → ; These images display that the nanocatalyst has a hexagonal structure. The average size of nanoparticles was established at around 12 nm.

### Usage of the synthesis Fe_3_O_4_@CPTMO-phenylalanine-Ni as a nanocatalyst for the preparation of pyrazole derivatives 4a-i

After the characterization of Fe_3_O_4_@CPTMO-phenylalanine-Ni, it is employed in the preparation of entitled fused pyrazoles through the one-pot, the three-component approach involving arylglyoxals (1 mmol), cyclic 1,3-diketones (1 mmol) and 3-methyl-1-aryl-1*H*-pyrazole-5-amines (1 mmol) as precursors in the presence of different amounts of magnetic nanocatalyst. To optimize the reaction conditions, the reaction between phenylglyoxal (**1a**), cyclopentane-1,3-dione (**2a**), and 3-methyl-1-phenyl-1*H*-pyrazole-5-amines (**3a**) with a molar ratio of 1:1:1 was specified as a standard example reaction. At the outset, the example reaction was probed comprehensively in different conditions involving the effect of several solvents, catalyst loading, time, and reaction yield (Table 2). It should be noted that in the reaction performance in the absence of a nanocatalyst, no progress in the reaction was monitored (Table 2, entry 1). In the presence of 30 mg of catalyst, the reaction is performed with high speed and high efficiency, which affords the corresponding product in 85%, yield (Table 2, entry 2). Then, the effect of various types of solvents, such as acetone, ethanol, and n-hexane, was examined (Table 2, entries 3–8). As can be seen in Table 2, the best conditions for the reaction were obtained in water/acetone (2:1) as a green and sustainable solvent and 30 mg of the nanocatalyst, which was done in less than 3 min, and the reaction efficiency was quite satisfying (Table 1, entry 5). As an outcome, using higher and lower amounts of nanocatalyst (40 and 20 mg) had not affected the yield (Table 2, entries 8 and 4, respectively).

**Table 2:** Effect of some parameters (solvent and amount of catalyst) for the preparation of **4a**.


Entry	Catalyst (mg)	Solvent	Temperature (°C)	Time (min)	Yield (%)
1	Fe_3_O_4_@CPTMO-phenylalanine-Ni (0)	Water	Reflux	720	0
2	Fe_3_O_4_@CPTMO-phenylalanine-Ni (30)	Water	Reflux	25	85
3	Fe_3_O_4_@CPTMO-phenylalanine-Ni (30)	Acetone	Reflux	30	80
4	Fe_3_O_4_@CPTMO-phenylalanine-Ni (20)	Water/Acetone (2:1)	80	30	88
5	Fe_3_O_4_@CPTMO-phenylalanine-Ni (30)	Water/Acetone (2:1)	80	3	98
6	Fe_3_O_4_@CPTMO-phenylalanine-Ni(30)	Ethanol	Reflux	45	70
7	Fe_3_O_4_@CPTMO-phenylalanine-Ni (30)	n-Hexane	Reflux	60	43
8	Fe_3_O_4_@CPTMO-phenylalanine-Ni (40)	Water/Acetone (2:1)	80	30	90
9	Fe_3_O_4_ (30)	Water/Acetone (2:1)	80	300	68
10	Fe_3_O_4_@CPTMO (30)	Water/Acetone (2:1)	80	250	78
11	Fe_3_O_4_@CPTMO-phenylalanine (30)	Water/Acetone (2:1)	80	180	82

Also, the efficiency of Fe_3_O_4_, Fe_3_O_4_@CPTMO, and Fe_3_O_4_@CPTMO-phenylalanine as catalyst was checked in model reaction indicating that those could produce the product (Table 2, entries 9–11). The obtained result demonstrates that Fe_3_O_4_@CPTMO-phenylalanine-Ni as nanocatalyst has the best catalytic performance compared to other catalyst, which can be attributed to the combination of organic, inorganic groups and metal.

After adequately evaluating, pleasantly, the mentioned reaction was tolerant for various arylglyoxals **1a-f**, different cyclic 1,3-dicarbonyls **2a-e**, 3-methyl-1-aryl-1*H*-pyrazole-5-amines **3a-c** as starting material and using Fe_3_O_4_@CPTMO-phenylalanine-Ni in refluxing water/acetone (2:1) as an efficient pathway afforded a series of desired pyrazoles **4a-i.** The TON (turnover number) and TOF (turnover frequency) of the Fe_3_O_4_@CPTMO-phenylalanine-Ni were also estimated based on the amount of the loaded nickel in the catalyst (determined by ICP), exhibiting high catalytic activity leading to high yields of the desired products (Table 3).

**Table 3:** Preparation of fused pyrazoles **4a-i** catalyzed by Fe_3_O_4_@CPTMO-phenylalanine-Ni.


Entry	X	Y	Z	Product	Time (min)	Yield^a^(%)	TOF^b^ (min^−1^)	Reference
1	H 4-Br 4-Cl 4-F 4-Me 4-NO_2_		H		3	97	24.87	Poursattar Marjani et al.^34^
2			H		4	91	17.5	Poursattar Marjani et al.^34^
3			H		5	93	14.31	Poursattar Marjani et al.^34^
4			H		7	98	10.77	Polo et al.^35^
5			3-Cl		4	97	18.65	Polo et al.^35^
6			4-Cl		3	95	24.36	Polo et al.^35^
7			H		4	92	17.69	Polo et al.^35^
8			3-Cl		5	91	14	Polo et al.^35^
9			4-Cl		3	97	24.87	Polo et al.^35^

Conditions: Arylglyoxals (1 mmol), cyclic 1,3-diketones (1 mmol), 3-methyl-1-aryl-1*H*-pyrazol-5-amines (1 mmol), catalyst (30 mg) and H_2_O/acetone (10 mL), 3–7 min.

^a^Isolated yield.

^b^TOF = TON/time (min), TON = Yield (%)/(mol%, Cat, based on ICP).

**Figure 11:**
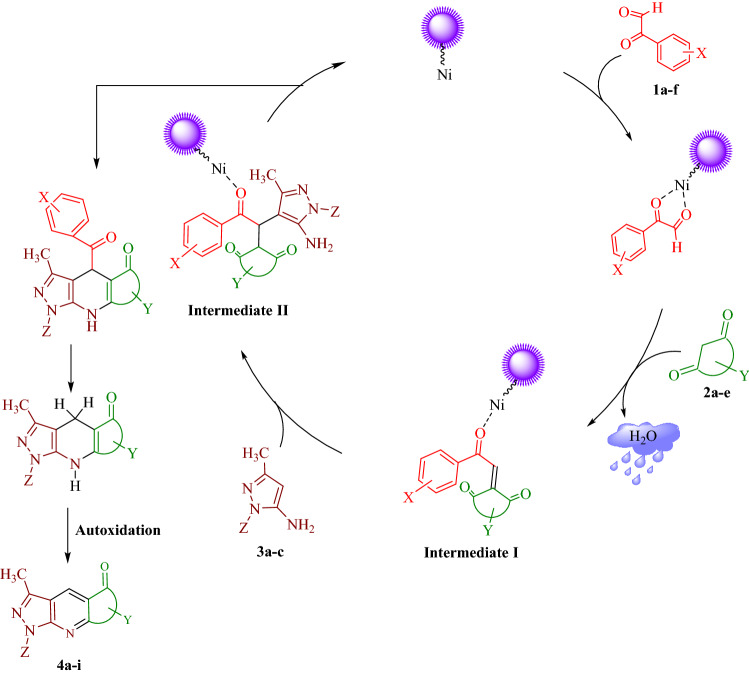
The possible reaction mechanism for the obtaining of compounds** 4a-i**.

Figure 11, offers a probable mechanistic rationalization in forming compounds **4a-i** via three-component domino reactions, catalyzed by Fe_3_O_4_@CPTMO-phenylalanine-Ni. It first postulated that coordination between oxygen atoms in carbonyl groups of arylgloxals **1a-f** and Ni of catalyst occurred. Simultaneously, in situ *Knoevenagel* condensation of cyclic 1,3-dicarbonyls **2a-c** with activated formyl group of arylglyoxals **1a-f**, by expulsing a water molecule to form the benzididine compound (intermediate **I**). Following *Michael’s* addition of benzididine compound to the substrate, **3a-c** resulted in the intermediate to the reaction cycle. Finally, open-chain intermediate **II** performs an intramolecular heterocyclization, dearoylation, and subsequent autoxidation process leading to the formation of the compounds **4a-i** and regenerating catalyst in the reaction mixture. Indeed, the new catalyst is essential to accelerating and facilitating the reaction cycle.

Also, the catalytic activity of this synthesized magnetic nanocatalyst was compared with previously explored procedures for the formation of pyrazole derivatives is demonstrated in Table 4. As clearly revealed in Table 4, the Fe_3_O_4_@CPTMO-phenylalanine-Ni possesses especial benifits, and acted convincingly superior over the reported methods concerning the reaction time and yield.

**Table 4:** Comparison of the effect of various catalysts' efficiency in the formation of compound **4b**.

Entry	Catalyst	Conditions	Time (min)	Yield (%)	Reference
1	InCl_3_	MW/heating	15	82	Polo et al.^35^
2	L-proline	H_2_O/reflux	60	87	Sumesh et al.^36^
3	Acetic acid	Reflux	120	76	Dzvinchuk^37^
4	Fe_3_O_4_@CPTMO	Water/Acetone	250	78	This work
5	Fe_3_O_4_@CPTMO-phenylalanine	Water/Acetone	180	82	This work
6	Fe_3_O_4_@CPTMO-phenylalanine-Ni	Water/Acetone	3	98	This work

### Hot filtration

**Figure 12:**
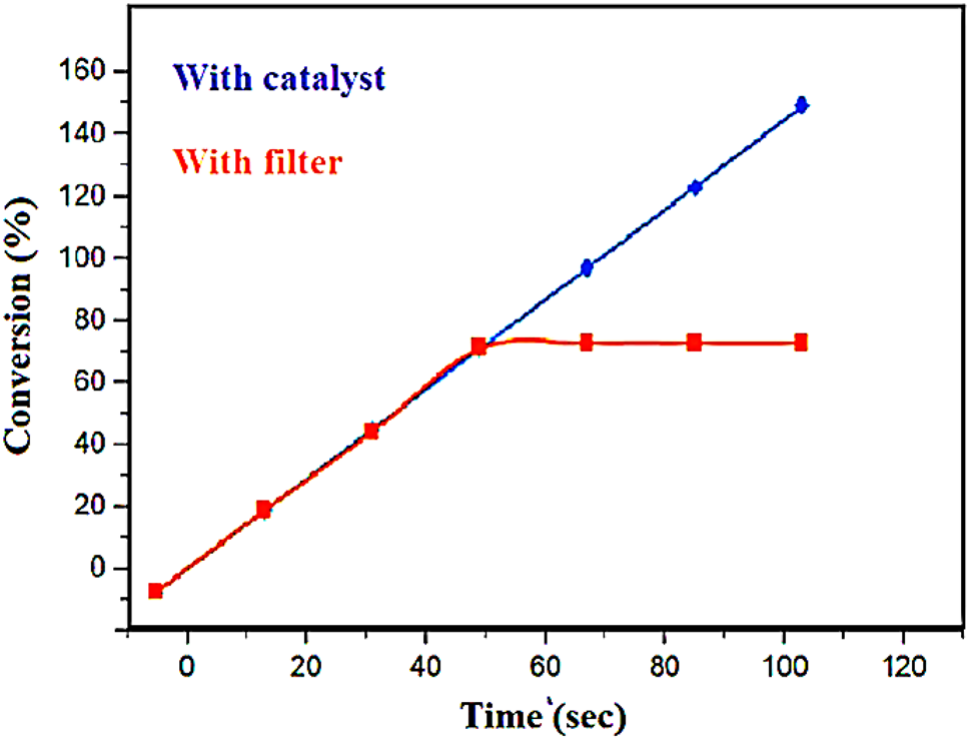
Hot filtration of Fe_3_O_4_@CPTMO-phenylalanine-Ni.

To experiment with the degree of nickel saturation in the obtained nanocatalyst, the synthesis of pyrazole derivatives was carried out in the middle of the reaction. When the reaction progressed to 50%, the catalyst was removed from the reaction, and it was observed that there was no progress, and it remained at the same efficiency as before (Fig. 12). Based on these observations, it can be said that the catalyst acts heterogeneously and the reaction does not take place in the solution phase.

### Renewability

**Figure 13:**
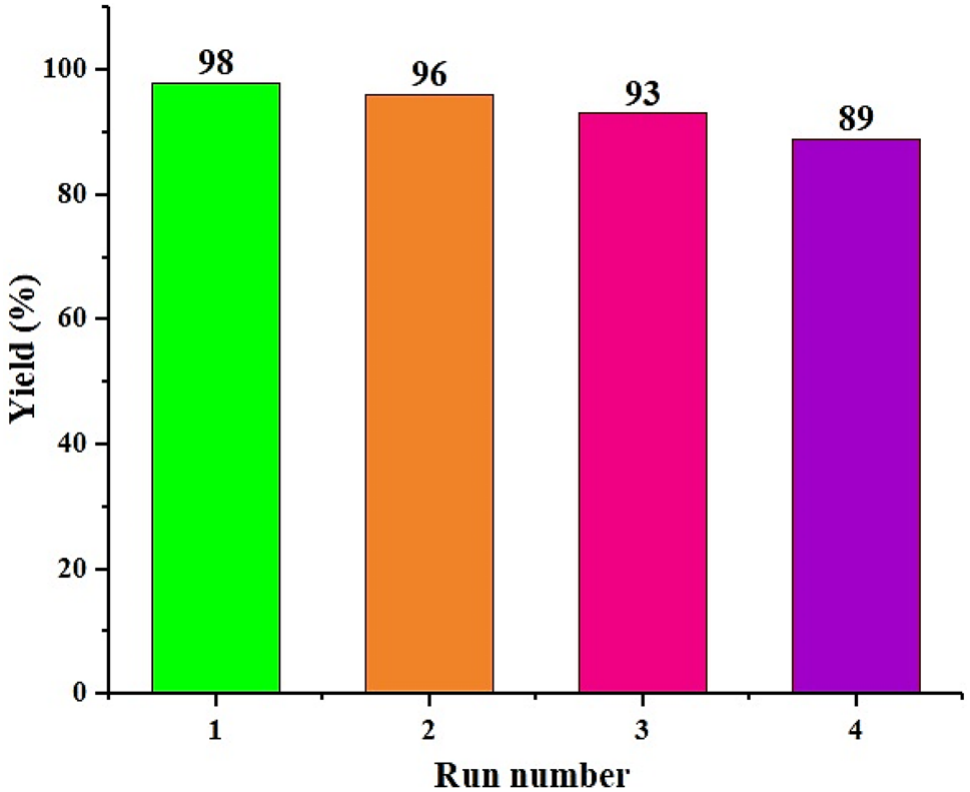
Recycling of Fe_3_O_4_@CPTMO-phenylalanine-Ni.

The renewability of catalysts in commercial reactions has great importance, so at this stage, the reproducibility of the catalyst has been investigated. So that after the reaction was completed in each step, the nanocatalyst was eliminated by an outside magnet bar and several were washed with diethyl ether. This process has continued up to 4 stages, and the activity of the catalyst is retained even after four repeated usages (Fig. 13). According to ICP-EOS measurements, the Ni content was 1.19% after the start of the reaction, which reached 1% after the washing steps. Under the analysis of SEM, the particle size was 20 nm before the start of the reaction, which reached 33 nm after the completion of 4 reaction steps.

## Experimental

### Instrument and materials

All starting substances utilized in the experiment section were obtained from Fluka and Sigma-Aldrich and were used without refining. The PEG used in this reaction had a molecular weight of 400. FT-IR spectra were measured on a Thermo-Nicolet Nexus 670 spectrometer. TLC was accomplished to control the development of the reaction with aluminum plate coating. The morphology and the elemental analysis of the nanocatalyst were tested using FESEM and EDS analysis (HITECH S-4160). TGA was registered using the Mettler Stare SW 9.10 instrument. TEM analysis was performed by the CM30, Philips device. A vibration sample magnetometer (VSM) with a Lake Shore VSM 7410 was used to identify the magnetic properties of the sample. The surface area, pore volume, and average particle size were taken with the Brunauer–Emmett–Teller (BET) and the Barrett-Joyner-Halenda (BJH) analysis. Both ^1^H-NMR at 300.13 MHz and ^13^C-NMR at 75.5 MHz spectra were estimated on a Bruker spectrometer.

### Preparation of Fe_3_O_4_/PEG nanoparticle (A)

To a mixture of FeCl_3_.6H_2_O (2 g) and FeCl_2_.4H_2_O (1 g) (a molar ratio; 2:1) in distilled water (30 mL) was added a solution of ammonium hydroxide (25%, 10 mL) and obtained a black solution. After that, it was placed at room temperature for 9 h until dried. In the next step, the black precipitate with the amount of sodium dodecyl sulfate (SDS, 0.1 g) in water (30 mL) was placed in an ultrasonic device in the mixture for 30 min. Next, PEG-400 (12 mL) was put in the ultrasonic device for 1 h. The obtained material was rinsed with water/ethanol and dried (at 97 °C for 10 h).

### Preparation of Fe_3_O_4_@CPTMO-phenylalanine (B)

At the outset, a mixture of 3-chloropropyl-trimethoxysilane (CPTMO, 5 g) in n-hexane (30 mL) was dropwise added into the precipitate of the previous step and refluxed under nitrogen gas at 100 °C, for 24 h. Then obtaining material was dried under a vacuum for 6 h at 67 °C. After that, phenylalanine (1 g), EtOH (25 mL), and Et_3_N (1.5 mL) were added to the reaction medium and placed under reflux conditions at 70 °C for 12 h. Finally, the product was dried for 12 h in an oven.

### Preparation of Fe_3_O_4_@CPTMO-phenylalanine-Ni (C)

In the final step, Ni (NO_3_)_2_.6H_2_O (500 mg) in absolute ethanol (25 mL) was added to compound **B (**900 mg**)**, the mixture was placed under reflux for 12 h, and then dried at 78 °C, and a novel catalyst was achieved.

### General method of synthesis of fused pyrazoles 4a-i

A mixture of substituted arylglyoxals (**1a-f**, 1 mmol), cyclic 1,3-dicarbonyls (**2a-e**, 1 mmol), and 3-methyl-1-aryl-1*H*-pyrazole-5-amines (**3a-c**, 1 mmol) in water/acetone (molar ratio, 2:1, 5 mL), and Fe_3_O_4_@CPTMO-phenylalanine-Ni nanoparticles (30 mg) were prepared. The obtained mixture was stirred at 80 °C for suitable times (Table3, reaction time in the range of 3–7 min). The reaction progress was assayed with TLC. Upon completion of the reaction, the nanocatalyst was effortlessly isolated from the final product using an external magnetic field and rinsed with water/acetone, and dried, after that, used more times in the reactions. Eventually, the solvent was evaporated from the product mixture, and the residue was filtered off by suction to collect the relevant pyrazole derivatives in high overall yields.

## Conclusion

In this current research, Fe_3_O_4_@CPTMO-phenylalanine-Ni recyclable, heterogeneous, and magnetic nanocatalyst was successfully synthesized. FT-IR, TGA, SEM, ICP, XRD, EDS, BET, VSM, and TEM analyses were used to identify this nanocatalyst. To evaluate the activity and application of the synthesized magnetic nanocatalyst, the synthesis reaction of substituted pyrazoles was designed, and this reaction was investigated in the presence of the synthesized nanocatalyst in mild reaction conditions. The noteworthy results and prominent advantages of this research are a shorter reaction time, excellent product yields, ease of handling, more straightforward procedures, quick set-up, no-unwanted products, and easy separation of catalyst. Due to the magnetic properties of the synthesized nanocatalyst, it can be retrieved and reutilized, which was used for at least four runs after the completion of the desired reaction without a significant decrease in catalytic activity.

## Supplementary Information

Supplementary Information.

## Data Availability

All data generated or analyzed during this study are included in this published article [and its supplementary information files].
