# Supplementary material for: Synthesis of new magnetic nanocatalyst Fe3O4@CPTMO-phenylalanine-Ni and its catalytic effect in the preparation of substituted pyrazoles
Source: Sci Rep. 2023 Feb 13;13:2564. doi: 10.1038/s41598-023-29598-6 (PMC9925813; doi:10.1038/s41598-023-29598-6)
Supplement: Supplementary file 1 — Supplementary Information. [file 41598_2023_29598_MOESM1_ESM.pdf]

Supporting Information

Synthesis of new magnetic nanocatalyst Fe_3O_4@CPTMO-phenylalanine-Ni and its catalytic effect in the preparation of substituted pyrazoles

Samaneh Bikas, Ahmad Poursattar Marjani*, Sepideh Bibak & Hamideh Sarreshtehdar Aslaheh
Department of Organic Chemistry, Faculty of Chemistry, Urmia University, Urmia, Iran
*E-mail: a.poursattar@urmia.ac.ir; a.poursattar@gmail.com

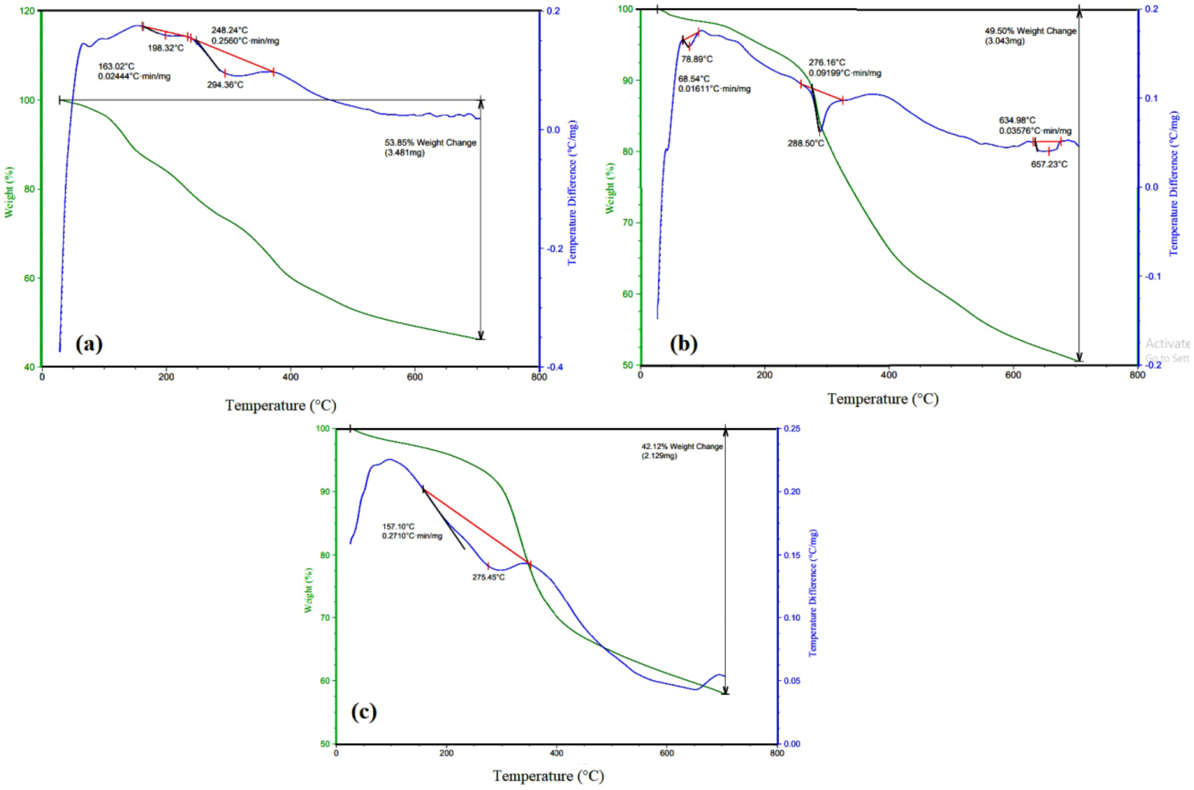

Figure S1. TGA thermogram and definitions of differential thermal analysis (DTA) of Fe_3O_4@CPTMO-phenylalanine-Ni (**a**), Fe_3O_4@CPTMO-phenylalanine (**b**) and Fe_3O_4@CPTMO (**c**).

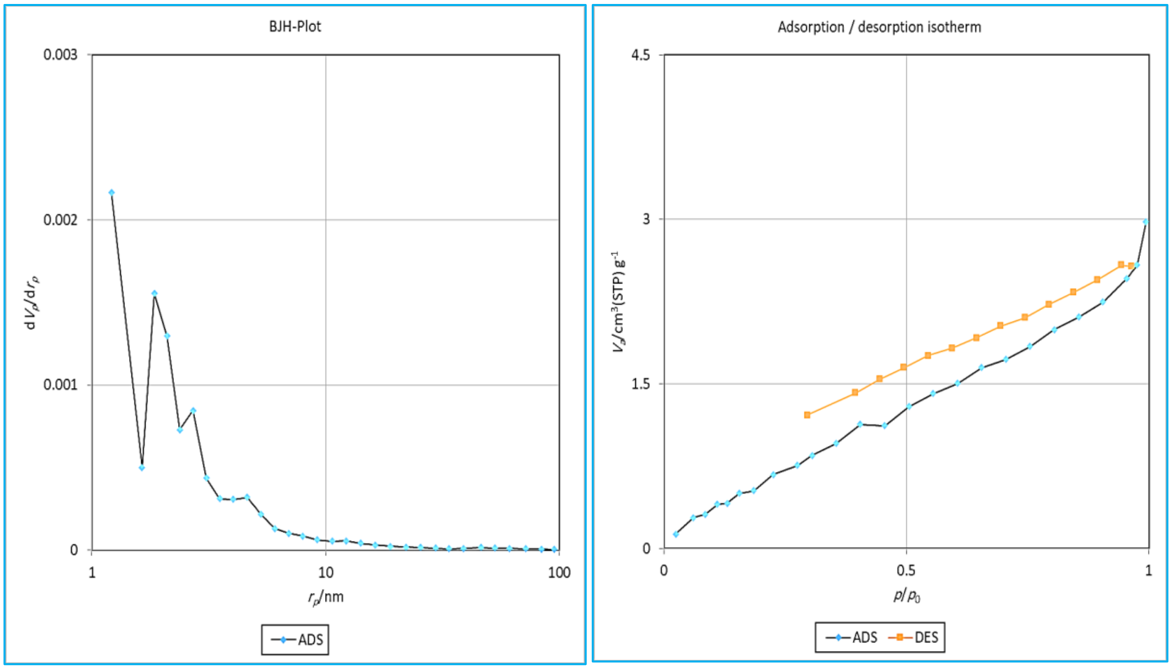

Figure S2. Nitrogen adsorption/desorption isotherms (left) and pore size distributions (right) of nanocatalyst.

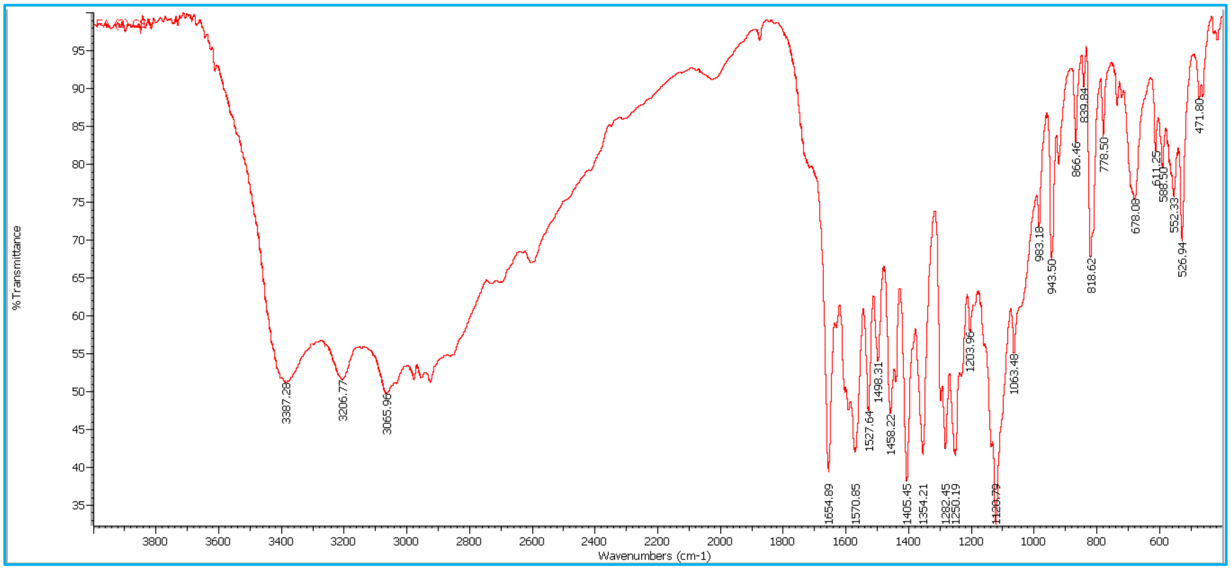

FT-IR spectrum of recovered Fe_3O_4@CPTMO-phenylalanine-Ni

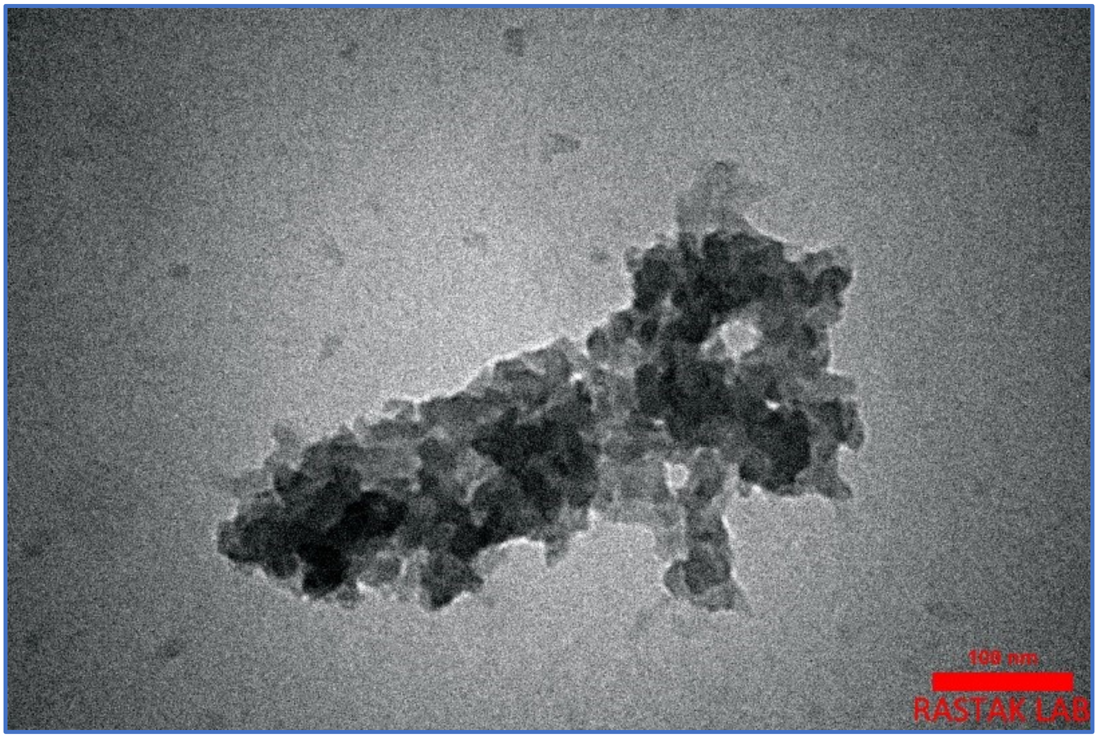

TEM image of recycled Fe_3O_4@CPTMO-phenylalanine-Ni

3-Methyl-1-phenyl-6,7-dihydrocyclopenta[*b*]pyrazolo[4,3-*e*]pyridin-5(1*H*)-one (4a).

^{1}H-NMR ($CDCl_3$) δ (ppm): 2.32 (s, 3H, CH_3), 2.88–2.83 (m, 2H, CH_2), 3.43–3.47 (m, 2H, CH_2), 7.41 (t, 1H, J = 7.5 Hz, ArH) 7.57 (t, 2H, J = 8.1 Hz, ArH), 7.78 (d, 2H, J = 8.4 Hz, ArH), 8.36 (s, 1H, ArH). IR (KBr, cm^{-1}): 3435, 2945, 2877, 1680, 1593, 1502, 1479, 1416, 1381, 1326, 1263, 1222, 1182, 1014, 911, 879, 837, 757, 688.

3-Methyl-1-phenyl-1,6,7,8-tetrahydro-5*H*-pyrazolo[3,4-*b*]quinolin-5-one (4b).

^{1}H-NMR ($CDCl_3$) δ (ppm): 2.25 (quin, J = 6.3 Hz, 2H, CH_2), 2.67 (s, 3H, CH_3), 2.77 (t, 2H, J = 6.3 Hz, CH_2), 3.29 (t, 2H, J = 6.3 Hz, CH_2), 7.31 (t, 1H, J = 7.2 Hz, ArH), 7.53 (t, 2H, J = 7.8 Hz, ArH), 8.31 (d, 2H, J = 7.8 Hz, ArH), 8.77 (s, 1H, ArH). IR (KBr, cm^{-1}): 3344, 3029, 2955, 1661, 1616, 1527, 1459, 1363, 1189, 1119, 1066, 1003, 847, 754.

3,8,8-Trimethyl-1-phenyl-1,6,7,8-tetrahydro-5*H*-pyrazolo[3,4-*b*]quinolin-5-one (4c).

^{1}H-NMR ($CDCl_3$) δ (ppm): 1.16 (s, 6H, 2×CH_3), 1.57 (bt, 2H, CH_2), 2.26 (bt, 2H, CH_2), 2.57 (s, 3H, CH_3), 7.33 (t, 1H, J = 7.2 Hz, ArH), 7.52 (t, 2H, J = 7.2 Hz, ArH), 8.27 (d, 2H, J = 8.1 Hz, ArH), 8.74 (s, 1H, ArH). IR (KBr, cm^{-1}): 3438, 3347, 3067, 2958, 2933, 2869, 1682, 1593, 1504, 1480, 1417, 1379, 1269, 1234, 1121, 1090, 1012, 979, 821, 779, 750, 684.

3,7,7-Trimethyl-1-phenyl-1,6,7,8-tetrahydro-5*H*-pyrazolo[3,4-*b*]quinolin-5-one (4d).

^{1}H-NMR ($CDCl_3$) δ (ppm): 1.15 (s, 6H, 2×CH_3), 2.62 (s, 2H, CH_2), 2.66 (s, 3H, CH_3), 3.17 (s, 2H, CH_2), 7.31 (t, 1H, J = 9 Hz, ArH), 7.53 (d, 2H, J = 9 Hz, ArH), 8.30 (d, 2H, J = 9 Hz, ArH), 8.73 (s, 1H, ArH). IR (KBr, cm^{-1}): 3432, 2951, 1678, 1569, 1592, 1494, 1415, 1375, 1281, 1243, 1116, 1022, 758, 679.

1-(3-Chlorophenyl)-3,7,7-trimethyl-1,6,7,8-tetrahydro-5*H*-pyrazolo[3,4-*b*]quinolin-5-one (4e).

^{1}H-NMR ($CDCl_3$) δ (ppm): 1.16 (s, 6H, 2×CH_3), 2.64 (s, 2H, CH_2), 2.67 (s, 3H, CH_3), 3.20 (s, 2H, CH_2), 7.26 (bd, overlapped by $CDCl_3$ impurity peak, 1H, ArH), 7.46 (t, 1H, J = 7.5 Hz, ArH), 8.32 (d, 1H, J = 7.5 Hz, ArH), 8.45 (s, 1H, ArH), 8.74 (s, 1H, ArH). IR (KBr, cm^{-1}): 3101, 2955, 2872, 1682, 1590, 1478, 1453, 1379, 1274, 1239, 1140, 1092, 898, 871, 778, 741, 679, 553.

1-(4-Chlorophenyl)-3,7,7-trimethyl-1,6,7,8-tetrahydro-5*H*-pyrazolo[3,4-*b*]quinolin-5-one (4f).

^{1}H-NMR ($CDCl_3$) δ (ppm): 1.16 (s, 6H, 2×CH_3), 2.64 (s, 2H, CH_2), 2.67 (s, 3H, CH_3), 3.18 (s, 2H, CH_2), 7.47 (d, 2H, *J* = 8.7 Hz, ArH), 8.32 (d, 2H, *J* = 8.7 Hz, ArH), 8.74 (s, 1H, ArH). IR (KBr, cm^{-1}): 3105, 2957, 2931, 2869, 1679, 1594, 1576, 1499, 1475, 1446, 1381, 1270, 1240, 1218, 1171, 1089, 1012, 829, 691, 556, 503.

3-Methyl-1-phenylindeno[1,2-*b*]pyrazolo[4,3-*e*]pyridin-5(1*H*)-one (4g).

^{1}H-NMR ($CDCl_3$) δ (ppm): 2.29 (s, 3H, CH_3), 7.40 (t, 1H, *J* = 6.9 Hz, ArH), 7.50 (t, 2H, *J* = 7.5 Hz, ArH), 7.60 (t, 1H, *J* = 7.8 Hz, ArH), 7.67 (t, 1H, *J* = 6.9 Hz, ArH), 7.89 (d, 2H, *J* = 7.8 Hz, ArH), 8.00 (d, 1H, *J* = 8.1 Hz, ArH), 8.28 (d, 1H, *J* = 7.2 Hz, ArH), 8.42 (s, 1H, ArH). IR (KBr, cm^{-1}): 3429, 3088, 2958, 2867, 1689, 1580, 1556, 1470, 1314, 1232, 1164, 1058, 946, 846, 759, 678, 592.

1-(3-Chlorophenyl)-3-methylindeno[1,2-*b*]pyrazolo[4,3-*e*]pyridin-5(1*H*)-one (4h).

^{1}H-NMR ($CDCl_3$) δ (ppm): 2.29 (s, 3H, CH_3), 7.35 (d, 1H, *J* = 7.8 Hz, ArH), 7.48 (s, 1H, ArH), 7.50 (t, 1H, *J* = 7.5 Hz, ArH), 7.68 (t, 1H, *J* = 6.9 Hz, ArH), 7.70 (t, 1H, *J* = 7.8 Hz, ArH), 7.86 (d, 1H, *J* = 7.5 Hz, ArH), 8.04 (d, 1H, *J* = 7.5 Hz, ArH), 8.28 (d, 1H, *J* = 8.4 Hz, ArH), 8.44 (s, 1H, ArH). IR (KBr, cm^{-1}): 3429, 3098, 2968, 2878, 1711, 1670, 1588, 1559, 1481, 1435, 1316, 1243, 1188, 1088, 1006, 946, 846, 764, 725, 676, 592.

1-(4-Chlorophenyl)-3-methylindeno[1,2-*b*]pyrazolo[4,3-*e*]pyridin-5(1*H*)-one (4i).

^{1}H-NMR ($CDCl_3$) δ (ppm): 2.50 (s, 3H, CH_3), 7.04 (d, 2H, *J* = 7.2 Hz, ArH), 7.36 (d, 2H, *J* = 7.8 Hz, ArH), 7.51 (t, 1H, *J* = 8.1 Hz, ArH), 7.63 (d, 1H, *J* = 8.1 Hz, ArH), 7.82 (t, 1H, *J* = 8.1 Hz, ArH), 8.13 (d, 1H, *J* = 8.1 Hz, ArH), 8.84 (s, 1H, ArH). IR (KBr, cm^{-1}): 3430, 3088, 2928, 1721, 1678, 1580, 1561, 1488, 1318, 1244, 1189, 1086, 944, 846, 765, 728, 595.
